# Concordance between nurse-reported quality of care and quality of care as publicly reported by nurse-sensitive indicators

**DOI:** 10.1186/s12913-016-1372-z

**Published:** 2016-04-06

**Authors:** Dewi Stalpers, Renate A. M. M. Kieft, Dimitri van der Linden, Marian J. Kaljouw, Marieke J. Schuurmans

**Affiliations:** St. Antonius Academy, St. Antonius Hospital Nieuwegein/Utrecht, PO Box 2500, 3430 EM Nieuwegein, The Netherlands; Dutch Nurses’ Association, PO Box 8212, 3503 RE Utrecht, The Netherlands; Institute of Psychology, Erasmus University, PO Box 1738, 3000 DR Rotterdam, The Netherlands; Dutch Health Care Authority, PO Box 3017, 3502 GA Utrecht, The Netherlands; Department of Revalidation, Nursing Science & Sports, University Medical Centre Utrecht, PO Box 85500, 3508 GA Utrecht, The Netherlands

**Keywords:** Hospitals, Nurse perception, Nursing care, Quality assessment, Quality indicators, Quality of care

## Abstract

**Background:**

Nurse-sensitive indicators and nurses’ satisfaction with the quality of care are two commonly used ways to measure quality of nursing care. However, little is known about the relationship between these kinds of measures. This study aimed to examine concordance between nurse-sensitive screening indicators and nurse-perceived quality of care.

**Methods:**

To calculate a composite performance score for each of six Dutch non-university teaching hospitals, the percentage scores of the publicly reported nurse-sensitive indicators: screening of delirium, screening of malnutrition, and pain assessments, were averaged (2011). Nurse-perceived quality ratings were obtained from staff nurses working in the same hospitals by the Dutch Essentials of Magnetism II survey (2010). Concordance between the quality measures was analyzed using Spearman’s rank correlation.

**Results:**

The mean screening performances ranged from 63 % to 93 % across the six hospitals. Nurse-perceived quality of care differed significantly between the hospitals, also after adjusting for nursing experience, educational level, and regularity of shifts. The hospitals with high-levels of nurse-perceived quality were also high-performing hospitals according to nurse-sensitive indicators. The relationship was true for high-performing as well as lower-performing hospitals, with strong correlations between the two quality measures (*r*_S_ = 0.943, *p* = 0.005).

**Conclusions:**

Our findings showed that there is a significant positive association between objectively measured nurse-sensitive screening indicators and subjectively measured perception of quality. Moreover, the two indicators of quality of nursing care provide corresponding quality rankings. This implies that improving factors that are associated with nurses’ perception of what they believe to be quality of care may also lead to better screening processes. Although convergent validity seems to be established, we emphasize that different kinds of quality measures could be used to complement each other, because various stakeholders may assign different values to the quality of nursing care.

## Background

Nursing care quality is important, because it is linked to patient safety, patient satisfaction, and other health care outcomes [1, 2]. However, assessing a multi-faceted concept such as quality of care has many challenges. Quality indicators are commonly used measures to gain insight into health care organizations’ performance regarding the quality of care provided. With regard to nursing quality, nurse-sensitive indicators are used, defined as “those outcomes that are relevant, based on nurses’ scope and domain of practice, and for which there is empirical evidence linking nursing inputs and interventions to the outcome for patients” [3, 4]. Health care systems across the world use the public reporting of these indicators for benchmarking purposes. Transparency of quality is of great importance for informed decision-making by various stakeholders, such as health care providers, consumers, insurance companies and policy makers [5]. As in other countries, all hospitals in the Netherlands annually have to report on a mandatory set of nurse-sensitive indicators. Since 2007, the Dutch Health Care Inspectorate requires hospitals to publicly report indicators, such as delirium, malnutrition, pain and pressure ulcers [6].

In the literature, there is much debate about the reliability and validity of nurse-sensitive indicators. For example, studies by Doran and colleagues [7], and Maas and colleagues [8] showed that nurses are able to collect reliable data regarding indicators (e.g., pain). On the other hand, the need for methodological checks of indicators as accurate measures of quality is also emphasized by various authors [9–11]. To contribute to the existing literature about nurse-sensitive indicators, the aim of the present study is to explore the convergent validity of these quality indicators by examining the correspondence with a nurse-reported measure of quality, namely nurses’ perception of the quality of care. Where nurse-sensitive indicators provide a quantitative basis to monitor and evaluate nursing care and are referred to as objective quality measures, nurse-reported measures are used to determine nurses’ perceptions and are referred to as subjective quality measures [12].

Regarding the objective measures, our focus is on nurse-sensitive screening indicators, referring to how often patients’ risk identification has taken place after admission to the hospital. Screening of health risks is one of the core duties of nurses and therefore well-suited as an indicator of care quality [13]. Furthermore, screening indicators are relatively easy to obtain and hospitals can be compared based on their performance without the complex task of adjusting for differences in patients’ risks in the various hospitals [14]. We investigated data from six non-university teaching hospitals in the Netherlands. We examined: (i) the performance of each hospital on the following nurse-sensitive screening indicators: delirium, malnutrition, and pain assessments, (ii) nurses’ perception of the quality of care; and whether any statistical differences between the hospitals can be ascribed to differences in nurse characteristics, and (iii) whether there is concordance between the two measures of quality of nursing care.

## Methods

### Study design and sample

This cross-sectional study included data from staff nurses working in one of six non-university teaching hospitals located in different parts of the Netherlands. In the Dutch health care setting, teaching hospitals are general hospitals with a transcending regional role and a teaching status. These hospitals are not equal to academic hospitals, as in many other countries (e.g., USA, Canada), because the university based faculty and a specific research role are not present [15]. The data concerning hospital characteristics, such as hospital size (number of licensed beds) and nursing full-time equivalents (FTE) were supplied by the hospital organizations themselves and the Dutch Hospital Association.

### Nurses’ perception of quality of care

In the year 2010, the Dutch Nurses’ Association issued the Dutch version of the Essentials of Magnetism II survey (D-EoM II) to all contracted staff nurses of the six hospitals. The D-EoM II survey, a validated instrument, asks nurses questions about their work environment, quality of care in their department, job satisfaction, and demographic characteristics [16, 17]. In this study, we used the scores from the question regarding nurse-perceived quality of care: ‘On a scale of 1 to 10, with 1 representing ‘dangerously low quality’ and 10 representing ‘very high quality’, how do you rate the quality of patient care in your own hospital unit?’ The overall response rate to the survey was 53.3 % and 2338 nurses (=46.8 %) answered all the questions, including the nurse-perceived quality of care score.

We included the following demographic characteristics of nurses: (i) experience, (ii) education level, and (iii) working shift. Experience in nursing was expressed in years and was categorized per 5 years, ranging from less than 5 years to over 30 years. Nurses’ education level was defined as: (i) Registered Nurses (RNs) with an Associate’s degree in nursing, (ii) RNs with a Bachelor’s degree in nursing, and (iii) RNs with a Bachelor’s degree and additional training; with differences regarding complexity of roles and degree of responsibilities [18]. Working shift referred to the kinds of shifts that nurses work, including: (i) fixed shifts (i.e., exclusively day shifts, evening shifts or night shifts), and (ii) rotating shifts. We did not include the effect of gender, because the sample almost exclusively consisted of women. We also decided to exclude age from the analyses, because the years of experience were strongly co-related to age.

### Nurse-sensitive indicators

The national database of the Dutch Health Care Inspectorate was used to obtain nurse-sensitive indicator data. At the end of each year, all Dutch hospitals use their internal data management systems to extract the previously defined and legislated quality indicators. The data are publicly reported on a website (www.ziekenhuizentransparant.nl). In this study, the 2011 dataset was used, including five nurse-sensitive screening indicators concerning delirium, malnutrition, and pain [19]. The definitions and data collection methods are presented in Table 1.

**Table 1:** Definitions of nurse-sensitive screening indicators

Indicators	Definition by numerator-denominator	Data collection
Screening of delirium	Number of hospital units in which a risk score was included in the medical record for more than 80 % of all patients 70 years and older	Collected yearly from hospital unit-based data management systems. Submitted to the Inspectorate yearly by hospital organizations.
	Total number of hospital units with admitted patients 70 years and older	
Observation of delirium	Number of patients observed at least once using the measuring methods of DOSS or CAM for the presence of delirium, regardless of the outcome	Collected daily from hospital unit-based data management systems. Submitted to the Inspectorate yearly by hospital organizations.
	Total number of patients with an increased risk of delirium (‘screening of delirium’)	
Screening of malnutrition	Number of adult patients which on admission are screened for malnutrition	Collected daily from hospital unit-based data management systems. Submitted to the Inspectorate yearly by hospital organizations.
	Total number of clinically admitted adult patients in a year	
Standardized pain assessment in post-operative patients in the recovery room	Number of clinical post-operative patients with a standardized pain assessment in the recovery room	Collected daily from hospital unit-based data management systems. Submitted to the Inspectorate yearly by hospital organizations.
	Total number of clinical post-operative patients in the recovery room	
Standardized pain assessment in post-operative patients in hospital units	Number of clinical post-operative patients with a standardized pain assessment in hospital units	Collected daily from hospital unit-based data management systems. Submitted to the Inspectorate yearly by hospital organizations.
	Total number of clinical post-operative patients in hospital units	

Inspectie voor de Gezondheidszorg. Kwaliteitsindicatoren. Basisset ziekenhuizen 2011 [19]

### Ethical statement

This research was executed in compliance with the Helsinki Declaration. The Dutch Hospital Data (DHD) reviewed the study protocol in accordance with the protocol ‘DHD-databases use’ and with local regulations in the Netherlands (Data Protection Act), and gave formal approval to conduct the study (reference number 12.11.21.01/PH.sdh.). Nurses’ participation in the survey study was voluntary and anonymous. It was mentioned to them that completing and submitting the survey automatically meant that they gave informed consent.

### Statistical analysis

First, descriptive statistics were used to characterize the staff nurses in our sample. To test differences in quality scores among stratified groups of nurses, we used analysis of variance (ANOVA) with Bonferroni post-hoc tests (adjusting for multiple comparisons). The assumptions of normally distributed data were met by normality plots of this large sample. We used univariate general linear models (GLM) to analyze differences in perceived quality between the six hospitals; adjusting for the nurse characteristics (experience, education level, working shifts) by including them into the model simultaneously.

To categorize nurse-perceived quality of care, we determined the percentage of satisfied nurses per hospital; the higher the percentage, the higher hospitals’ performance. Nurses who gave a quality score of ≥ 8 (on a scale from 1 to 10) were labeled ‘very satisfied’, ‘satisfied’ refers to ≥ 6-8 and ‘not satisfied’ refers to < 6. Additionally, we ranked the hospitals ranging from 1^st^ to 6^th^, in which the ranking value of 1^st^ represents the highest-performing hospital (i.e., hospital with the highest percentage of satisfied and very satisfied nurses). We considered nurse-perceived quality of care as a subjective measure regarding nursing quality (i.e., influenced by the nurse’s personal judgment).

Regarding nurse-sensitive indicators, we calculated a composite score to address each of the six hospitals’ performance level. A valid and simple method to compose a composite score is by averaging percentages [20, 21]. The percentages on the five screening indicators, as described by numerator and denominator in Table 1, were used for this purpose. The composite scores for each hospital were used to categorize the quality of hospitals; the higher the percentage, the higher hospitals’ performance. We ranked the hospitals ranging from 1^st^ to 6^th^, in which the ranking values of 1^st^ resembles the highest-performing hospital (i.e., hospital with the highest mean composite score). We considered nurse-sensitive indicators as objective measures of nursing quality (i.e. involving an impartial measurement, that is, without bias or prejudice).

To test the association between the objective indicators of care and nurses’ perception of care, we took the mean composite hospital score on the indicators and correlated that with the percentage of satisfied nurses per hospital. Due to the fact that these analyses were conducted at the hospital-level, we used Spearman’s Rho correlation which is the appropriate method in this context as it is known to compare differences in rank-order. All statistical analyses were conducted using SPSS version 22.

## Results

The characteristics of nurses and the six hospitals are shown in Table 2. Nursing experience ranged between 1 and 40 years, with an average of 16.8 years across the sample. Predominantly nurses had at least a Bachelor’s degree (64.9 %) and were working rotating shifts (80.6 %). The majority of hospitals were mid-sized; there were two larger hospitals, with more than 1000 licensed beds and more than 1000 nursing FTE.

**Table 2:** Demographic characteristics of the study sample

	Licensed beds	Nursing FTE	Nurses	Experience		Education level						Working shifts^a^			
						Associate		Bachelor		Bachelor+		Fixed		Rotating	
	N	N	N	Mean	SD	N	%	N	%	N	%	N	%	N	%
All hospitals			2338	16.76	11.13	821	35.1	1131	48.4	386	16.5	447	19.4	1862	80.6
Hospital A	1102	1198	452	16.54	11.50	221	48.9	177	39.2	54	11.9	112	24.8	337	74.6
Hospital B	663	808	314	18.12	10.50	119	37.9	146	46.5	49	15.6	70	22.3	237	75.5
Hospital C	696	964	326	14.63	10.90	123	37.7	159	48.8	44	13.5	52	16.0	272	83.4
Hospital D	580	795	348	18.49	11.34	133	38.2	146	42.0	69	19.8	61	17.5	282	81.0
Hospital E	1070	1143	595	17.80	11.00	171	28.7	336	56.5	88	14.8	68	11.4	519	87.2
Hospital F	555	813	303	13.94	10.65	54	17.8	167	55.1	82	27.1	84	27.7	215	71.0

Bachelors + are RNs with a Bachelor’s degree and additional training

^a^Missing values regarding working shifts: All hospitals (*N =* 29), Hospital A (3), Hospital B (7), Hospital C (2), Hospital D (5), Hospital E (8), Hospital F (4)

The mean perceived quality scores for the hospitals ranged from 6.61 (*SD* = 1.24) to 7.11 (*SD* = 1.09). There was a strong positive correlation between years of experience and nurse-perceived quality; more experienced nurses were significantly more satisfied than less experienced nurses. Additionally, nurses with 20 to 25 years of experience were most satisfied, followed by nurses with 25 and 30 or more years of experience. RNs with an Associate’s degree were significantly less satisfied as compared to RNs with a Bachelor’s degree. Regarding working shifts, it was shown that nurses working fixed shifts were more satisfied than nurses working rotating shifts. Nurses working dayshifts were most satisfied with the quality of care in their hospital. The differences between the six hospitals were significant [*F*(5, 2332) = 8.397; *p* <0.01] and post-hoc tests revealed that Hospital C had a significantly lower mean score, as opposed to the other hospitals. These differences could not be attributed to nurse characteristics (experience, education and working shifts), because after controlling for these characteristics the effects remained significant [*F*(5, 2284) = 3.011; *p* =0.01].

Table 3 summarizes nurses’ perception of quality of care and the ranking of the six hospitals. The majority of nurses were satisfied with the quality of care in their hospital. Approximately 9 % (*N* = 219) were not satisfied and rated the quality of their hospital unit with a score less than 6. Table 3 indicates that, based on the percentage of satisfied (quality score ≥ 6-8) and very satisfied nurses (quality score ≥ 8), Hospital D had the best results and Hospital C had the least favorable results.

**Table 3:** Ranking by nurses’ perception of quality of care

	All	Hospital A	Hospital B	Hospital C	Hospital D	Hospital E	Hospital F
	*N =* 2338	*N =* 452	*N =* 314	*N =* 326	*N =* 348	*N =* 595	*N =* 303
Nurse-perceived quality of care							
% Not satisfied <6 (*N*)	9.4 (*219*)	10.2 (*46*)	9.6 (*30*)	16.3 (*53*)	6.6 (*23*)	7.4 (*44*)	7.6 (*23*)
% Satisfied ≥6-8 (*N*)	58.9 (*1377*)	58.8 (*266*)	57.3 (*180*)	62.3 (*203*)	55.7 (*194*)	62.4 (*371*)	53.8 (*163*)
% Very satisfied ≥8 (*N*)	31.7 (*742*)	31.0 (*140*)	33.1 (*104*)	21.5 (*70*)	37.6 (*131*)	30.3 (*180*)	38.6 (*117*)
Ranking							
% Satisfied + very satisfied	90.6	89.8	90.4	83.8	93.3	92.7	92.4

Table 4 shows the results regarding the nurse-sensitive indicators. High screening percentages were shown for the indicators of pain; in particular ‘pain assessment in the recovery room’, with values ranging from 90 to 100 %. Large differences between hospitals were found for the screening indicators of malnutrition and delirium; in particular ‘observation of delirium’, with values between 15 and 100 %. Based on the mean composite scores, Hospital D was identified as the highest-performing hospital with a composite score of 93.2 % and Hospital C had the least favorable composite score of 62.9 %.

**Table 4:** Ranking by nurse-sensitive indicators

	Hospital A	Hospital B	Hospital C	Hospital D	Hospital E	Hospital F
Quality indicator^a^						
% Screening delirium	26.3	61.5	23.1	81.3	86.4	78.6
(*N* screened/total *N*)	*(5/19)*	*(8/13)*	*(3/13)*	*(13/16)*	*(19/22)*	*(11/14)*
% Observation delirium	79.8	51.7	32.2	91.9	100.0	15.0
(*N* observed/total *N*)	*(197/247)*	*(45/87)*	*(430/1337)*	*(91/99)*	*(425/425)*	*(9/60)*
% Screening malnutrition	45.7	82.0	81.4	94.8	78.6	82.0
(*N* screened/total *N*)	*(6439/14095)*	*(16683/20345)*	*(15175/18637)*	*(16483/17379)*	*(18468/23507)*	*(854/1042)*
% Pain recovery room	90.1	90.0	100.0	100.0	100.0	99.7
(*N* assessed/total *N*)	*(6418/7121)*	*(8087/8986)*	*(9473/9473)*	*(11775/11775)*	*(10595/10595)*	*(8432/8456)*
% Pain hospital units	83.7	99.4	78.0	98.1	97.1	59.0
(*N* assessed/total *N*)	*(13045/15583)*	*(8932/8986)*	*(7388/9473)*	*(1411/1439)*	*(10943/11272)*	*(4428/7505)*
Ranking						
Composite score	65.1	76.9	62.9	93.2	92.4	66.9

^a^Nurse-sensitive screening indicators (see definitions Table 1)

We assessed Spearman’s Rho correlations to test the overlap between nurse-perceived quality of care and nurse-sensitive indicators. A strong significant correlation was shown between the two quality measures of *r*_S_ =0.943 (*p* = 0.005). Hospitals’ ranking according to both measures of quality are shown in Table 5. There was a high degree of correspondence; nurses were generally most satisfied in hospitals with high scores on nurse-sensitive indicators, and least satisfied in lower-scoring hospitals.

**Table 5:** Ranking of quality of nursing care in six Dutch hospitals

	Subjectively measured quality	Objectively measured quality	Ranking nurse-perceived quality	Ranking nurse-sensitive indicators
Hospital A	89.8	65.1	5^th^	5^th^
Hospital B	90.4	76.9	4^th^	3^rd^
Hospital C	83.8	62.9	6^th^	6^th^
Hospital D	93.3	93.2	1^st^	1^st^
Hospital E	92.7	92.4	2^nd^	2^nd^
Hospital F	92.4	66.9	3^rd^	4^th^

Rank 1^st^ denotes the best result, and 6^th^ the least favorable result

## Discussion

Nurse-sensitive indicators are widely used to evaluate the quality of nursing care. The present study examines their convergent validity by investigating concordance between publicly reported nurse-sensitive screening indicators (delirium, malnutrition, pain) and nurse-reported quality of care. To our knowledge, this is one of the first studies to explore the direct relationship between objectively measured quality of nursing care and subjectively measured quality, from a nurses’ point of view. We found that there was a substantial correlation between the two quality measures. As such, our study adds knowledge to the international debate on the value of nurse-sensitive indicators as measures of quality of nursing care.

In literature, there is a scientific debate about the usefulness of publicly reported quality indicators as comparative performance measures. Critics claim that, because nurse-sensitive indicators are reported by hospital organizations themselves, there is a risk that they adjust the data in order to achieve goals of external accountability [10, 22]. On the other hand, there is evidence that public reporting is associated with actual quality of care [23, 24] and stimulates quality improvement activities at the hospital level [25]. In our study, we demonstrated that there is a strong relationship between publicly reported screening indicators and nurses’ satisfaction with the quality of care, thereby implicating that these indicators both can be used to assess nursing care quality. However, we emphasize that the two quality measures are not likely to be completely interchangeable. Needleman and colleagues [2] stated that various kinds of quality measures potentially could have their own value for stakeholders. For example, regarding nurse-sensitive indicators, policy makers and insurance companies could use screening indicators to benchmark hospitals and hospital units. Nurse-sensitive screening indicators are particularly suitable for these kinds of purposes, because they are easy to measure and screening activities are a prime task of nurses. Additionally, health care organizations (e.g., hospitals) may benefit more from satisfaction with care ratings, because they provide input for quality improvement in a specific setting. Thus, the optimal approach for defining quality of nursing care depends on the underlying question and who poses the question.

Comparing objective versus subjective measures is increasingly relevant in current health care research. Previous studies demonstrated significant associations between hospital performance and patient-perceived quality. For example, Jaipaul et al. [26] reported lower mortality rates in hospitals with higher patient satisfaction with overall quality, and Nelson et al. [27] found that hospitals’ financial performance was associated with patients’ perception of quality of care. With regard to nurse-perceived quality, some studies elaborated on the relationship with medical performance indicators. McHugh and Witkoski Stimpfel [28] examined the convergent validity of nurse-reported quality by analyzing the correspondence with composite scores for processes related to acute myocardial infarction, pneumonia, and surgical patients. They reported that a 10 % increase in nurses’ satisfaction with the quality of care was associated with a 0.6 to 2.0 point increase in composite performance scores. Tvedt et al. [29] found significant correlations between nurse-reported quality and survival probabilities after stroke or acute myocardial infarction. Despite their relevance, these studies solely focused on medical performances. They did not exclusively focus on quality related to nurse-specific indicators (i.e., nurse-sensitive screening indicators). Future research about the usefulness of nurse-sensitive indicators as quality measures can contribute to a better understanding of quality of nursing care.

Our results that Bachelor’s educated nurses and more experienced nurses were mostly satisfied about quality of care is the opposite of what previous studies found (e.g., [17, 30]). We do not have a reasonable explanation for these differences, and therefore more studies assessing educational level and years of experience in relation to nurses’ perception of quality should be performed. The kinds of shifts that nurses are working has not often been included as a nurse characteristic. We found that nurses working fixed shifts, especially day shifts were more satisfied that those working rotating shifts. An interpretation is that nurses working rotating shifts may have a fragmented perspective of the quality of care, because of the rotating shift schedule. According to our results, the differences between the individual hospitals could not be explained by the included nurse characteristics. There is ample evidence that other factors, such as leadership, autonomy and nurse-physician relationships are important in relation to nurse-perceived quality and other quality outcomes (e.g., [17, 31]). The influence of these kinds of work environment factors however, was not the main focus of the present study.

### Limitations

One of the limitations is that, due to missing values on indicators, we were not able to calculate a composite score for each of the six hospitals in 2010. As a result, the nurse-sensitive indicator data were derived in 2011, whereas the survey data of nurses were conducted in 2010. We tested intra-correlations for all nurse-sensitive screening indicators in the full population of 93 Dutch hospitals and found moderate correlations (*r* = 0.59 to *r* = 0.67) between the years 2010 and 2011. Therefore, we argue that the results of both years are comparable and adequately reflect the Dutch context. Further research in a larger sample is necessary to support out findings, because our study sample was limited to six hospitals. Second, critics claim that it may be more interesting to extract unit-level data instead of hospital-level data, because there may be unit characteristics (e.g., patient complexity, workload) that are influential [22, 32]. Many attempts are made worldwide to benchmark on the unit-level, for example by ways of longitudinal studies on specific indicators, such as patient falls [33, 34]. However, it takes years before these kinds of processes are adequately implemented; this is an ongoing process which deserves attention [2, 8]. Third, we used one single-item score to determine satisfaction with quality of care. Although these kinds of quality scores are important indicators of nurses’ perspectives, they also have their limits. In line with previous studies [35], it would be useful to further explore interrelations with other satisfaction scores (e.g., recommendation of own hospital, job satisfaction). Fourth, a possible limitation is that some might have reservations about composite scores based on percentages. As described before, is was shown previously that these kinds of composite scores are useful measures to evaluate process performance [20, 21].

## Conclusions

Nurse-sensitive quality indicators and nurse-reported quality of care can offer opportunities to differentiate hospitals in terms of quality of nursing care. Our results confirm that quality indicators correspond with nurses’ perception of quality, by revealing strong correlations between the objective measurements from publicly reported indicators and nurses’ perceived quality of care from a survey. This finding implies that both quality measures are valuable as indicators of hospital performance. Because there is no golden standard to determine nursing care quality, various quality measures could be used by stakeholders (policy makers, health care providers etc.) to complement each other. All in light of the overarching goal of provision of excellent quality of care to patients.

## Availability of data and materials

The data supporting the conclusions regarding nurse-perceived quality of care are property of the Dutch Nurses’ Association and are available on request to the Dutch Nurses’ Association. The dataset of the Dutch Health Care Inspectorate supporting the conclusions regarding nurse-sensitive quality indicators is publicly available at http://www.ziekenhuizentransparant.nl/.

## Abbreviations

D-EoM II: Dutch Essentials of Magnetism II
FTE: full-time equivalents
RNs: Registered Nurses
